# Supplemental arginine vasopressin during the resuscitation of severe hemorrhagic shock preserves renal mitochondrial function

**DOI:** 10.1371/journal.pone.0186339

**Published:** 2017-10-24

**Authors:** Carrie A. Sims, Guan Yuxia, Khushboo Singh, Evan C. Werlin, Patrick M. Reilly, Joseph A. Baur

**Affiliations:** 1 The Trauma Center at the University of Pennsylvania, Department of Surgery, Perelman School of Medicine, Philadelphia, PA, United States of America; 2 Penn Acute Research Collaboration (PARC), University of Pennsylvania, Philadelphia, PA, United States of America; 3 Department of Surgery, University of California, San Francisco, San Francisco, CA, United States of America; 4 Department of Physiology, Perelman School of Medicine, University of Pennsylvania, Philadelphia, PA, United States of America; Georgia Regents University Cancer Center, UNITED STATES

## Abstract

Arginine vasopressin (AVP), a hormone secreted by the posterior pituitary, plays a vital role in maintaining vasomotor tone during acute blood loss. We hypothesized that decompensated hemorrhagic shock is associated with decreased AVP stores and supplementation during resuscitation would improve both blood pressure and renal function. Using a decompensated hemorrhagic shock model, male Long-Evans rats were bled to mean arterial blood pressure (MAP) of 40mmHg and maintained until the MAP could not be sustained without fluid. Once 40% of the shed volume was returned in lactated Ringer’s (Severe Shock), animals were resuscitated over 60 minutes with 4x the shed volume in lactated Ringer’s (LR) or the same fluids with AVP (0.5 units/kg+ 0.03 units/kg/min). Animals (n = 6-9/group) were sacrificed before hemorrhage (Sham), at Severe Shock, following resuscitation (60R, 60R with AVP) or 18 hours post-resuscitation (18hr, 18hr with AVP). Blood samples were taken to measure AVP levels and renal function. Pituitaries were harvested and assayed for AVP. Kidney samples were taken to assess mitochondrial function, histology, and oxidative damage. Baseline pituitary AVP stores (30,364 ± 5311 pg/mg) decreased with severe shock and were significantly depressed post-resuscitation (13,910 ± 3016 pg/ml. p<0.05) and at 18hr (15,592 ±1169 pg/ml, p<0.05). Resuscitation with LR+AVP led to higher serum AVP levels at 60R (31±8 vs 79±12; p<0.01) with an improved MAP both at 60R (125±3 vs 77±7mmHg; p<0.01) and 18hr (82±6 vs 69±5mmHg;p<0.05). AVP supplementation preserved complex I respiratory capacity at 60R and both complex I and II function at 18hr (p<0.05). AVP was also associated with decreased reactive oxygen species at 60R (856±67 vs 622±48F RFU) and significantly decreased oxidative damage as measured by mitochondrial lipid peroxidation (0.9±0.1 vs 1.7±0.1 fold change, p<0.01) and nitrosylation (0.9±0.1 vs 1.4±0.2 fold change, p<0.05). With AVP, renal damage was mitigated at 60R and histologic architecture was conserved at 18 hours. In conclusion, pituitary and serum AVP levels decrease during severe hemorrhage and may contribute to the development of decompensated hemorrhagic shock. Supplementing exogenous AVP during resuscitation improves blood pressure, preserves renal mitochondrial function, and mitigates acute kidney injury.

## Introduction

Nearly 130,000 people die of unintentional injury annually in the United States.[1] Of those who survive the initial trauma, hemorrhagic shock accounts for the majority of potentially preventable deaths.[2] Although intense vasoconstriction is the normal response to hemorrhagic shock, it cannot be maintained indefinitely. With prolonged hemorrhagic shock, intense vasoconstriction will progress to vasodilation and catecholamine-resistant cardiovascular collapse.[3, 4] Identifying strategies to prevent or treat this state of “decompensated shock” could be lifesaving.

Recently, arginine vasopressin (AVP) has been investigated as an adjunct during the resuscitation of severe trauma.[5–10] Secreted by the posterior pituitary in response to hypotension, AVP is essential for maintaining vasomotor tone during hemorrhagic shock. In animals lacking AVP, even minor blood loss results in significant hypotension and low levels during prolonged hemorrhagic shock have been associated with the development of catecholamine-resistant hypotension.[11–15] Clinically, severely injured trauma patients demonstrate a high incidence of AVP deficiency with an increased need for vasopressor support, blood product transfusions and prolonged ICU care.[9, 16, 17]

In addition to its vasopressor effects, AVP influences cellular metabolism and may improve mitochondrial function.[18, 19] When cells are treated *in vitro*, AVP appears to modulate the formation of NADH, activate mitochondrial ATP synthesis and inhibit apoptosis.[20–23] The *in vivo* impact of AVP on mitochondrial function and cell survival following hemorrhagic shock, however, is unknown.

We hypothesized that hemorrhagic shock results in decreased pituitary AVP stores and exogenous supplementation during resuscitation would improve both blood pressure and organ function. Given the kidney is the most common organ to fail following hemorrhagic shock, we elected to investigate the impact of vasopressin on renal function.[24]

## Methods

### Experimental protocol

All animal procedures were approved by the Institutional Animal Care and Use Committee of the University of Pennsylvania and in accordance with the guidelines established by the National Institutes of Health. Male Long-Evans rats (250–300 g) were housed in a facility with constant temperature and humidity with a 12-hour light/dark cycle. Animals were allowed to acclimate at least 2 days before surgery and given access to food and water *ad libitum*. Using a well-validated decompensated hemorrhagic shock model [25], rats were anesthetized using vaporized isoflurane (2–4%) by mask and underwent placement of femoral vascular catheters (PE50, Braintree Scientific, Inc., Brain-tree, MA). Mean arterial pressure (MAP) was recorded throughout the experimental protocol (Digi-Med Signal Analyzers, Louisville, KY, USA). A 5-cm midline laparotomy was performed to simulate soft tissue trauma. All surgical sites were bathed in 1% lidocaine and closed in layers. Animals received 0.25% buprenorphine (0.05mg/kg, subcutaneously). Animals were then placed in a plexiglass restraining apparatus and allowed to emerge from anesthesia. Full recovery from anesthesia with normalization of blood pressure and return of motor function took approximately 30 minutes.

**Fig 1:**
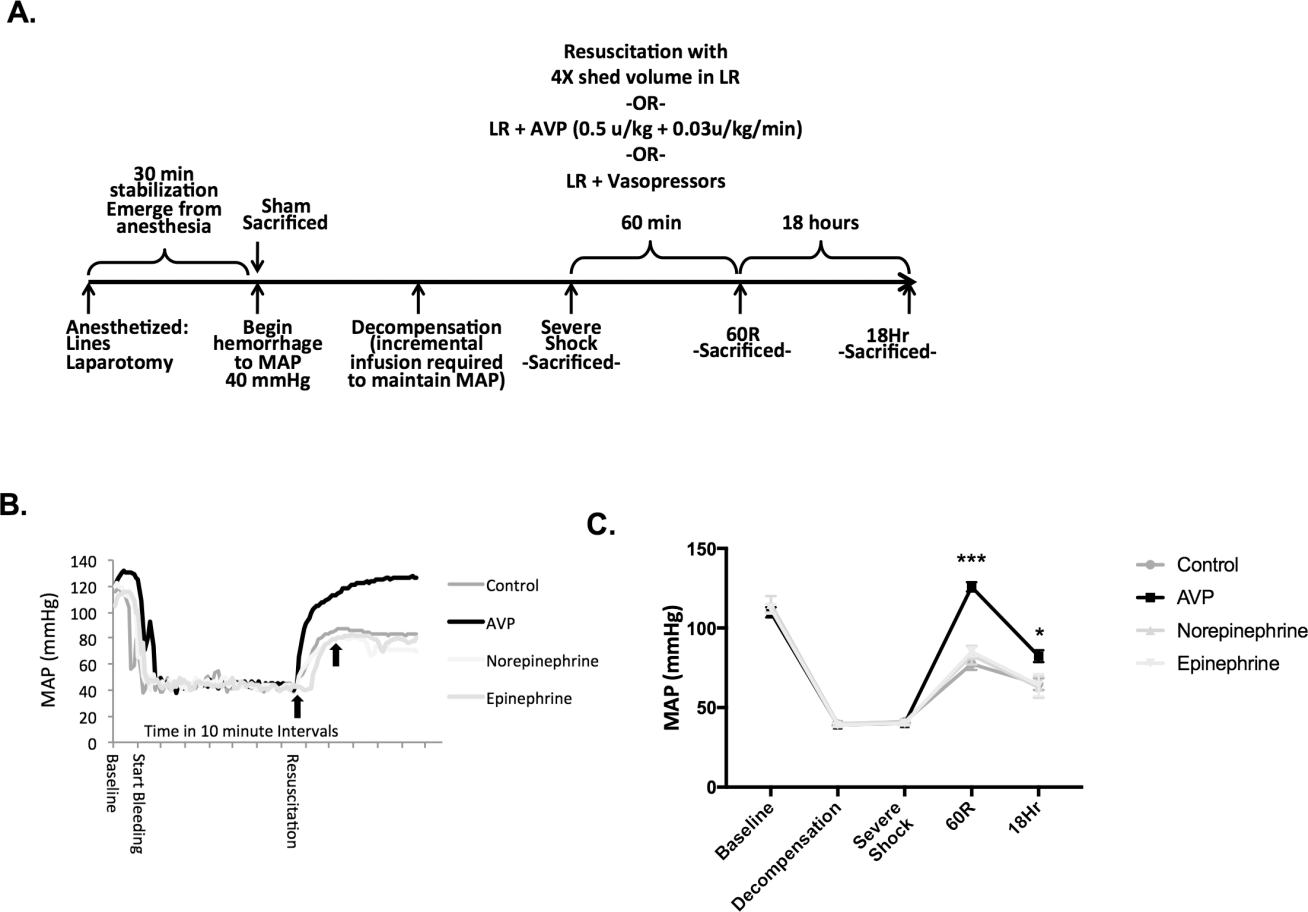
Resuscitation with AVP restores the mean arterial blood pressure. (**A**) Male Long Evans rats were anesthetized with isoflurane (4%) followed by vascular line placement and laparotomy to simulate soft tissue injury. After complete emergence from anesthesia, awake animals were passively bled to a mean arterial pressure (MAP) of 40 ± 5 mmHg. When animals could no longer maintain a MAP of 40 mmHg without fluid (Decompensation), they were given incremental 0.2 cc boluses of lactated Ringer’s (LR) until 40% of the shed volume was returned (Severe Shock). Animals were resuscitated over 60 minutes with 4X the shed volume in LR and either arginine vasopressin (AVP: 0.5 U/kg + 0.03 U/kg/hr) or placebo. Animals were sacrificed at Baseline (Sham n = 9/group), Decompensation (n = 9/group), Severe Shock (n = 9/group), following resuscitation (60R, n = 9/group) and 18 hours post resuscitation (n = 6/group). An additional group of animals were treated with increasing doses of norepinephrine (3 and then 6 ug/kg/min) or epinephrine (1.2 and then 2.4 ug/kg/min). (**B**) Blood pressure tracings of a representative animal from each group are depicted. Black arrows depict the time vasopressors were initiated, and then increased. (**C**) The mean arterial blood pressure (MAP) at each time point was recorded. Data were analyzed using one-way ANOVA with a post hoc Tukey’s test. Values are means ± SEM. AVP treated *vs*. other treatment groups; * *p*<0.05, ***p<0.001.

After full reversal of anesthesia and 30 minutes of hemodynamic stability, animals were randomized to 1 of 4 groups: Sham, Severe Shock, and Resuscitation with either lactated Ringer’s (LR) or LR + vasopressin (AVP, 0.5 units/kg units + 0.03 units/kg/min). All animals, except Shams, were passively bled via the femoral artery and maintained at a MAP of 40 ± 5 mmHg. When the blood pressure could no longer be maintained without fluid infusion (Decompensation), a MAP of 40 mmHg was sustained by incrementally infusing 0.2 cc boluses of LR. Animals were considered to be in Severe Shock when 40% of the total shed volume had been returned in the form of LR boluses. Animals were then resuscitated with four times the shed volume in LR with or without AVP (0.05 units/kg + 0.03 units/kg/min) over 60 minutes and followed for an additional 18 hours (Fig 1A). The use of a crystalloid only resuscitation has been used extensively in hemorrhagic shock models and avoids the potential transfusion of vasoactive substances, hormones, and cytokines found in the blood shed during hemorrhagic shock.[26–29] Animals (*n* = 6–9 per group) were anesthetized using isoflurane (2–4%) by mask at each time point (Sham, Severe Shock, 60 minutes (60R) and 18 hours post-resuscitation (18hr). A blood sample was assayed for arterial blood gases, lactate, hemoglobin, glucose, blood urea nitrogen (BUN) and electrolytes (i-STAT, Abbot Point of Care Inc., Princeton, NJ). Serum osmolality was calculated from i-STAT values (Osmolality = (2*Na) + BUN/2.8 + glucose /18). Serum creatinine was determined using a Vitros 350 blood biochemistry analyser (Ortho Clinical Diagnostics, Rochester, NY). Serum neutrophil gelatinase-associated lipocalin (NGAL) levels were measured post hoc by ELISA (Abcam, Cambridge, MA) according to the manufacturer’s directions. Bilateral kidneys were rapidly harvested following blood sampling. Animals were then euthanized using intravenous pentobarbital (150mg/kg) and immediately decapitated for pituitary extraction.

In a separate set of experiments, other adrenergic agonists were used during resuscitation as a control for the potential effect of vasopressin on mean arterial blood pressure. Animals (n = 3/group) were given increasing infusion of norepinephrine (3 and then 6 μg/kg/min) or epinephrine (1.2 and then 2.4 μg/kg/min) in addition to LR (4X the shed volume) during resuscitation.

### Vasopressin measurements

Whole pituitaries were immediately weighed and frozen in liquid nitrogen for storage. Frozen samples were homogenized in Walsh and Niall’s medium (15% trifluoroacetic Acid (TFA), 1% NaCl, and 5% formic Acid in 1M HCL) and stored at 4°C overnight. The sample was then filtered through glass wool 3 times and centrifuged at 15,000g for 20 minutes. The supernatant was passed through an acetonitrile/TFA prepared Sep-Pack C 18 column twice. AVP was eluted from the column using 5 ml of 80% acetonitrile and 0.1% TFA. After removing the acetonitrile under liquid nitrogen, the sample was desiccated under vacuum. Samples were reconstituted with assay buffer and the AVP concentration determined using a commercially available kit according to the manufacturer’s instructions (Arg8-Vasopressin ELISA, Enzo Life Sciences, UK)

Blood samples were collected into chilled EDTA tubes containing aprotonin (500 KIU/ml) and immediately centrifuged at 1,600g for 15 minutes. Plasma was stored at -70°C until samples could be processed as a single group. AVP was extracted from the plasma after thawing on ice by adding 2X the volume of ice-cold acetone. After vortexing, samples were centrifuged at 12,000g for 20 minutes. The supernatant was combined with 5X the volume of ice-cold petroleum ether and centrifuged at 10,000g for 10 minutes. The ether layer was discarded and the aqueous protein layer was desiccated under vacuum. As above, assay buffer was added, and the AVP concentration was measured using the Arg8-Vasopressin ELISA kit (Enzo Life Sciences, UK).

### Isolation of mitochondria

Whole kidneys (4–6 g) were excised and processed immediately at each time point. After quickly dissecting away connective tissue and fat, samples were weighed and immersed in ice- cold mitochondrial isolation buffer (MIB) (210 mM mannitol, 70 mM sucrose, 10 mM HEPES, 1 mM EDTA with final pH adjusted to 7.2 using KOH and supplemented with 0.5% fatty acid-free BSA). Kidneys were diced and homogenized with an additional 10 volumes (wt/vol) of MIB with 5% BSA using Potter Elvehjem homogenizer and a loose-fitting Teflon pestle. Mitochondrial isolation was performed using differential centrifugation as previously described.[30] The homogenate was centrifuged for 10 minutes at 1000g (4°C). The supernatant was collected and recentrifuged for 10 minutes at 9,600g. The pellet was resuspended in 15 ml MIB without BSA and centrifuged for an additional 10 minutes at 9,600g for further mitochondrial purification. The final mitochondrial pellet was resuspended in MIB and protein concentration was determined spectrophotometrically using the Biuret method with BSA as standard.

### Mitochondrial complex I (CI) and complex II (CII) respiratory capacity

Isolated mitochondria (1mg) were resuspended in respiration media (1 ml, 110mM mannitol, 0.5mM EGTA, 3mM MgCl_2_, 20mM taurine 10mM KH2PO4, 60mM K lactobionate, 0.3mM DTT, and 0.1% BSA (fatty acid free), adjusted to pH of 7.1 with KOH. Oxygen consumption was measured using a thermostatically regulated Clark oxygen electrode at 30°C (Hansatech Instruments, Norfolk, UK). Following stabilization (3–5 minutes), real-time oxygen concentration and flux data were collected continuously. After the basal respiration rate was recorded, complex I (CI)-dependent mitochondrial respiration was induced by adding 10 mM glutamate, 5 mM malate and 1 mM ADP.[31]

In a separate experiment, isolated mitochondria (1mg) were resuspended in respiration media and placed in the Hansatech instrument as described. Following stabilization, the basal respiration rate was recorded and complex II (CII)-dependent mitochondrial respiration was induced by adding rotenone (0.5μM), a selective CI inhibitor, 1 mM ADP and succinate (10mM).[31]

### Electron transfer from complex I (CI) to complex III (CIII)

Mitochondrial CI to CIII electron flow activity was measured in isolated mitochondrial samples as rotenone-sensitive NADH:cytochrome *c* reductase activity. Briefly, mitochondria were subjected to 2 freeze-thaw cycles (-70°C to 4°C) in order to break the membranes, 50 μg of mitochondria were added to a cuvette containing 50 μM oxidized cytochrome c as the final electron acceptor, 1 mM KCN, 1 μM rotenone, and 100 mM potassium phosphate (pH 7.2) in a thermocontrolled spectrophotometer (Hitachi U-2810, Singapore) at 30°C. A baseline of absorbance at 550 nm was recorded, and the reaction was initiated with 0.2 mM NADH as the electron donor. The increase in absorbance, corresponding to cytochrome *c* reduction, was continuously recorded.[32]

### Total production of mitochondrial-derived reactive oxygen species (ROS)

To analyze the total production of ROS, isolated mitochondria (10 μg) were suspended in 1 ml of buffer (250 mM sucrose, 20 mM 3-[N-morpholino] butane sulfonic acid, 10 mMTris-base, 100 μMPi [K], 0.5 mM Mg^2+^, pH 7.0; 30°C) containing CI substrates (malate/glutamate, 2.5/2.5 mM) with 10 μM H_2_DCFDA. Antimycin A (an inhibitor of CIII; 0.5 μM) was added to allow the production of ROS. After incubation at 30°C for 1 hour, the fluorescent signal from dichlorofluorescein (DCF; excitation 488 nm, emission 525 nm) was detected and quantified using a Modulus Microplate Reader (Turner Biosystems, Sunnyvale, CA).[31]

### Measure of mitochondrial permeability transition (MPT)

As previously described calcium uptake in freshly isolated kidney mitochondria was measured as an indicator of the susceptibility to undergo MPT.[33] Mitochondria (0.5mg) were suspended in potassium chloride (KCl) media (125 mM KCl, 2 mM K_2_HPO_4_, 1 mM MgCl_2_, and 20 mM HEPES, pH 7.0) containing 0.1 μM Calcium green-5N (a fluorescent calcium indicator dye), 5 mM succinate, 4 μM rotenone, 0.5 μM oligomycin and 0.2 mM ADP (37°C). Fluorescence was continuously monitored at using a Perkin-Elmer LS-3 fluorescence spectrometer equipped with a stirring device with the excitation and emission set at 506 nm and 532 nm respectively. Measurement of mitochondrial Ca^2+^ uptake was measured by successively adding known amounts of CaCl_2_ to the medium containing mitochondria until there was a burst of Calcium green-5N signal.

### Protein 4-hydroxynonenal and 3-nitrotyrosine analysis

Overall tissue damage by reactive oxygen and nitrogen species was assessed by measuring 4- hydroxynonenal and 3-nitrotyrosine using Western blot (Abcam, Cambridge, MA). Briefly, kidney protein lysates extracted in RIPA buffer (20 μg) were loaded in a 4–12% polyacrylamide gel and separated by electrophoresis (Invitrogen, San Diego, CA). Proteins were transferred onto a nitrocellulose membrane (Bio-Rad, Richmond, CA). After the membranes were blocked for 1 hour at room temperature (10 mmol/L Tris, 150 mmol/L NaCl, and 0.05% Tween-20 supplemented with 5% dry milk), they were incubated with the respective primary antibodies at 1:1000 dilution overnight at 4°C. After washing, membranes were incubated with peroxidase- linked donkey anti-rabbit or sheep anti-mouse IgG secondary antibodies (Amersham, Buckinghamshire, UK) at 1:5,000 dilution for 1 hour at room temperature. Signals were developed by enhanced chemiluminescence (PerkinElmer Life Sciences, Boston, MA). Blots were subsequently stripped and reblotted with VDAC (Abcam, Cambridge, MA) as a loading control. Bands were scanned, quantified by densitometry and normalized to VDAC controls using ImageJ software (National Institutes of Health, Bethesda, MD).

### Histology

Kidney tissues at the 18 hour time point were fixed in 4% paraformaldehyde for 24 hours and then washed in 70% ethanol. Tissues were subsequently embedded in paraffin and sectioned (5μm) for Haemotoxylin and Eosin (H&E) staining. Histological damage was assessed by an experienced histopathologist blinded to treatment group. Each slide was evaluated at 50 different sections at 10X and quantified using the Endothelial, Glomerular, Tubular, Interstitial (EGTI) scoring system. The scoring system consists of histological damage in 4 components: Endothelial, Glomerular, Tubular, and Interstitial and ranges from 0 (no damage) to 14 (severe damage) (Table 1).

**Table 1:** The EGTI histology scoring system.

Tissue component	Damage	Score
Endothelial	No damage	0
	Endothelial swelling	1
	Endothelial disruption	2
	Endothelial loss	3
Glomerular	No damage	0
	Thickening of Bowman capsule	1
	Retraction of glomerular tuft	2
	Glomerular fibrosis	3
Tubular	No Damage	0
	Loss of brush border in < 25% of tubular cells.	1
	Loss of brush border in > 25% of tubular cells. Thickened basal membrane.	2
	(Plus) Inflammation, cast formation, necrosis in up to 60% of tubular cells	3
	(Plus) Necrosis in more than 60% of tubular cells	4
Interstitial	No damage	0
	Inflammation, hemorrhage, in less than 25% of tissue	1
	(Plus) Necrosis in less than 25% of tissue	2
	Necrosis up to 60%	3
	Necrosis more than 60%	4

### Data analysis

Analyses were performed using SPSS (SPSS Inc., Armonk, NY). Data were analyzed using a one-way ANOVA with a post hoc Tukey’s test. A Mann Whitney U test was used to analyze differences in histologic grading at 18 hours. Results are presented as mean ± SEM. A *p* value of less than 0.05 was considered statistically significant.

## Results

### Physiologic and laboratory parameters

Blood pressure was maintained at a fixed MAP of 40 ± 5 mmHg during shock. Resuscitation with LR alone failed to restore the MAP to pre-shock values. Moreover, addition of increasing doses of norepinephrine or epinephrine failed to restore the MAP to baseline values at 60R. In contrast, the use of AVP during resuscitation, significantly improved the MAP at 60R and was similar to baseline values. Although the MAP was lower than baseline at 18 hours in both groups, AVP treated rats had a significantly higher blood pressure at this late time point (Fig 1B and 1C).

As expected, our decompensated hemorrhagic shock model resulted in severe lactic acidosis, hyperglycemia, and uremia. Although creatinine increased in both groups, levels were not elevated above normal values for rats (0.2–0.8 mg/dL) (Table 2). Compared to LR alone, however, resuscitation with AVP was associated with significantly lower NGAL values at 60R (273 ± 41 *vs*. 171 ± 21 ng/ml, *p*< 0.05) and less lactate production at 18hr (2.2 ± 0.3 *vs*. 1.0 ± 0.2 mmol/L, *p*< 0.05).

**Table 2:** Laboratory values.

			*60R*		*18Hr*	
*Characteristics*	Sham	Severe Shock	Control	AVP	Control	AVP
***Lactate (mmol/L)***	1.3 ± 0.3	19.0 ± 2.0^a^	6.8 ± 3.7^a^	4.9 ± 2.0^a^	2.2 ± 1.3^a^^,^^c^	1.0 ± 0.2
***pH***	7.45 ± 0.05	6.95 ± 0.12^a^	7.35 ± 0.10^a^	7.37 ± 0.05^a^	7.42 ± 0.09	7.48 ± 0.04
***PO2 (mmHg)***	174 ± 42	139 ± 32^a^	118 ± 35^a^	107 ± 11^a^	119 ± 37	95 ± 27
***PCO2 (mmHg)***	42 ± 5	14 ± 6^a^	31 ± 9^a^	34 ± 4^a^	39 ± 9	35 ± 4^a^
***HCO3^-^ (mmol/L)***	30 ± 3	3 ± 1 ^a^	17 ± 6^a^	19 ± 3^a^	25 ± 5^a^	26 ± 2^a^
***Na^+^ (mmol/L)***	136 ± 3	132 ± 4	141 ± 17	136 ± 2	137 ± 4	139 ± 3
***Glucose (mg/dL)***	219 ± 95	392 ± 110^a^	201 ± 94	167 ± 55	138 ± 27^a^	148 ± 15
***BUN (mg/dL)***	22 ± 4	24 ± 4	25 ± 2	25 ± 5	71 ± 20^a^	57 ± 32^a^
***Creatinine (mg/dL)***	0.29 ± 0.02	0.63 ± 0.03^a^	0.54 ± 0.03^a^	0.50 ± 0.02^a^	0.71 ± 0.09^a^	0.62 ± 0.05^a^
***Serum Osmolarity***	292 ± 2	293 ± 1	303 ± 10	290 ± 2	304 ± 3^a^	307 ± 5^a^
***NGAL (ng/ml)***	30 ± 5	90 ± 15^a^	273 ± 41^a^^,^^b^	171 ± 21^a^	690 ± 57^a^	678 ± 44^a^
***Hemoglobin (g/L)***	12.9 ± 1.5	6.0 ± 1.4^a^	5.0 ± 1.0^a^	5.6 ± 1.0^a^	5.1 ± 0.8^a^	4.9 ± 1^a^

^a^ p<0.05 *vs*. Sham

^b^ p<0.05 60R Control *vs*. AVP

^c^ p<0.05 18Hr control *vs* AVP

### Pituitary and serum vasopressin levels

**Fig 2:**
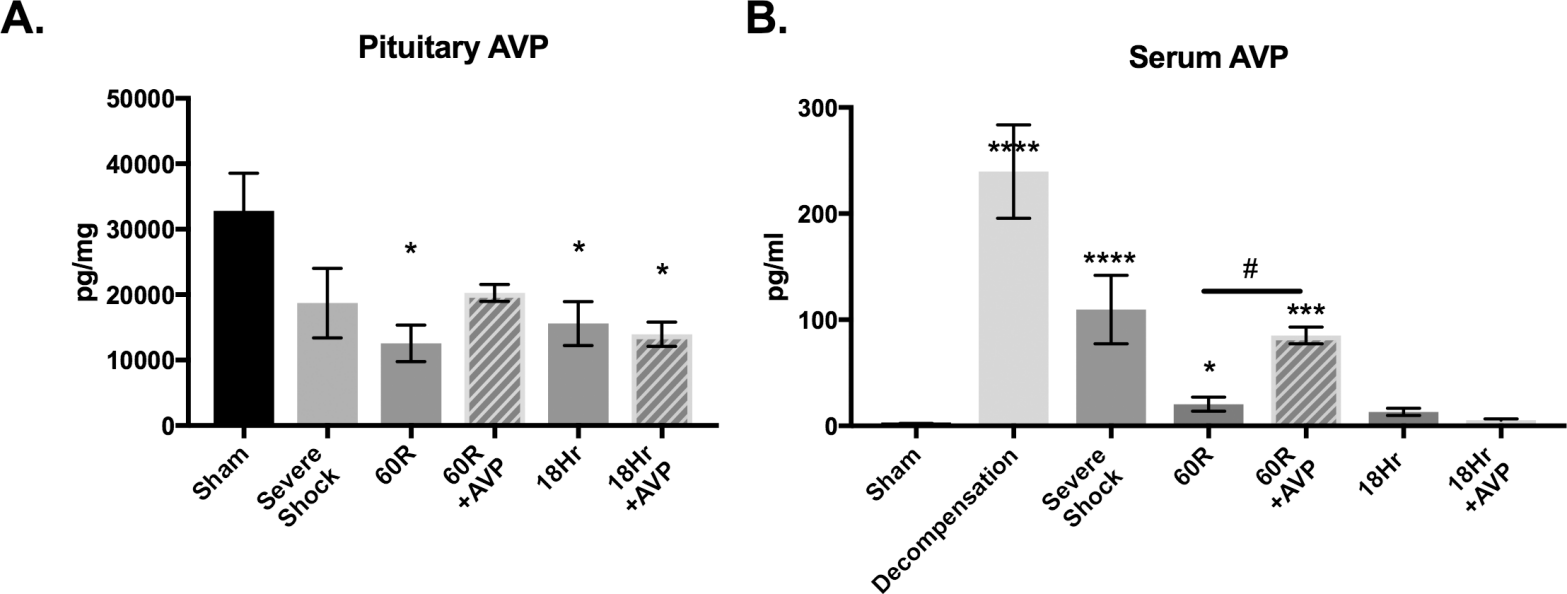
AVP levels decline despite persistent hypotension following hemorrhagic shock. Pituitary and serum AVP levels were sampled at baseline (Sham), Severe Shock, after 60 minutes of resuscitation (60R) and at 18 hours post resuscitation (n = 6–7 per time point). (**A**) Posterior pituitary levels (pg/ml ± SEM) of AVP decrease during hemorrhagic shock and were significantly depressed at both 60R and at 18 hours. (**B**) AVP serum levels (pg/ml ± SEM) appropriately increased but fell significantly following Decompensation. Data were analyzed using one-way ANOVA with a post hoc Tukey’s test. Values are mean ± SEM. Sham *vs*. other time points; *p<0.05, **p<0.01,***p<0.001, ****p<0.0001. AVP *vs*. Control; #p<0.05.

Decompensated hemorrhagic shock resulted in a depletion of pituitary AVP stores that continued to decline and became statistically significant at 18hr (27,688 ± 6732 pg/ml baseline vs. 8888 ± 1169 pg/ml 18hr) (Fig 2A). Serum vasopressin levels peaked at Decompensation, but decreased thereafter despite persistent hypotension in our control rats (Fig 2B). As expected, resuscitation with AVP significantly increased serum levels and mitigated the decline in pituitary AVP stores at 60R (Fig 2A and 2B).

### Mitochondrial function

**Fig 3:**
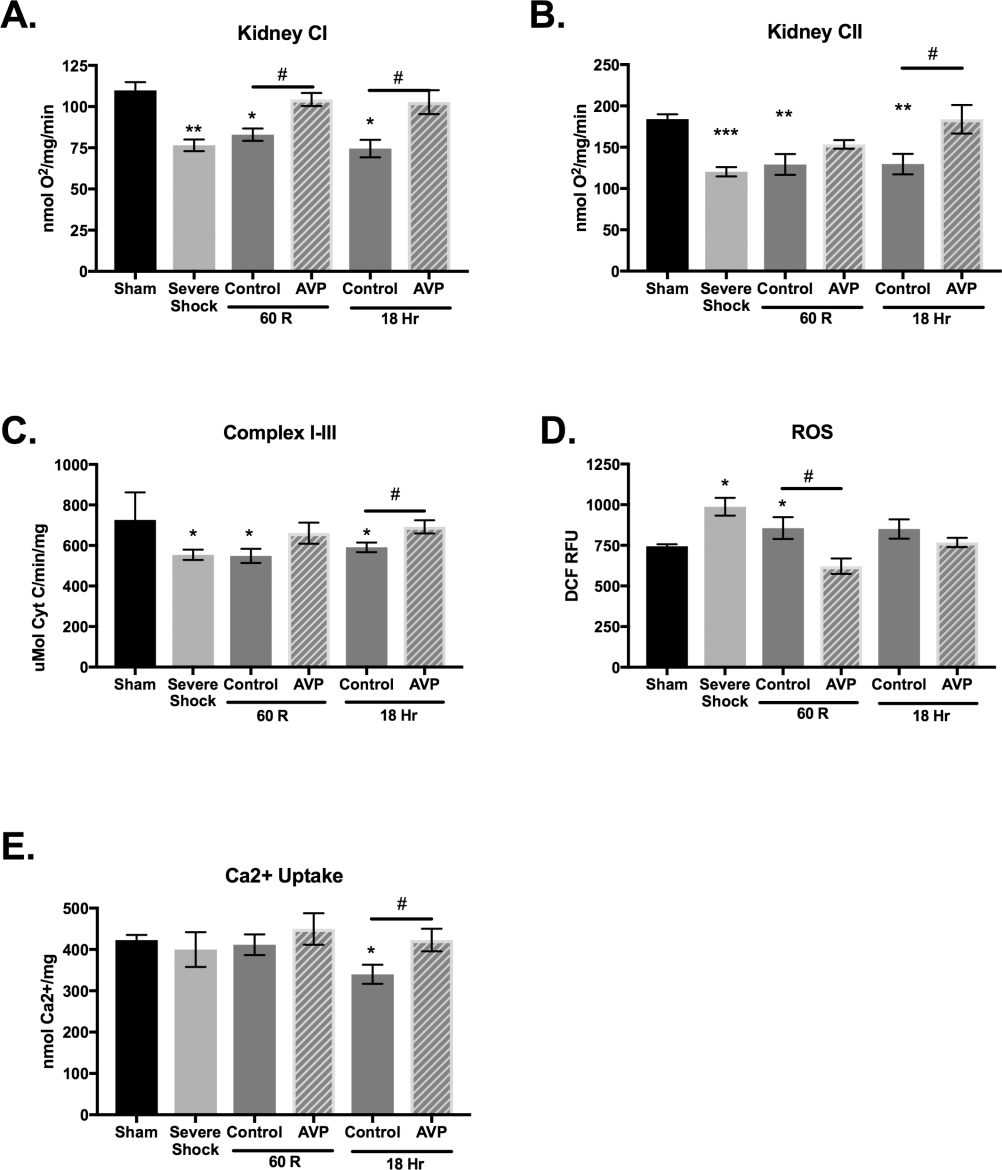
AVP supplementation during hemorrhagic shock is associated with improved mitochondrial function. (**A**) Complex I (CI) and (**B**) Complex II (CII) dependent respiratory capacity in isolated intact mitochondria was assessed by respirometry. Resuscitation with lactated Ringer’s (Control) resulted in impaired CI and CII respiration at 60 minutes and 18 hours. Addition of AVP (0.5u/kg bolus + 0.03u/kg/hr) during resuscitation preserved CI respiration and improved CII dependent respiration. (**C)**. The electron transport from CI to Complex III (CIII) was measured spectrophotometrically in isolated mitochondrial membranes. While control animals demonstrated impaired electron transfer, AVP supplementation preserved CI to CIII electron transfer. (**D**) The production of radical oxygen species (ROS) in isolated mitochondria was measured using the fluorescent signal from dichlorofluorescein (DCF). The increase in ROS following resuscitation (60R) was mitigated with AVP. (**E**) Mitochondrial stability was assessed by measuring calcium uptake as a marker of mitochondrial permeability transition. At 18 hours post resuscitation, control mitochondria were less stable than mitochondria isolated from AVP resuscitated animals. N = 6–7 animals per time point. Values are mean ± SEM. Data were analyzed using one-way ANOVA with a post hoc Tukey’s test. *p<0.05 *vs*. Sham; #*p*<0.05 Control *vs*. AVP treated.

Resuscitation with AVP preserved mitochondrial function both acutely and 18 hours post resuscitation. Following hemorrhagic shock and resuscitation with LR, mitochondrial respiration and electron transfer capability were significantly depressed (Fig 3A–3C). Resuscitation with AVP, however, completely mitigated the decline in CI-dependent respiration at 60R and 18hr. The AVP treated group also demonstrated improved CII-dependent respiration and electron transfer capability at 18hr. Given the improved CI function observed with AVP, it was not surprising that mitochondria harvested from AVP treated animals at 60R had significantly decreased production of free radical oxygen species (Fig 3D). Lastly, resuscitation with AVP resulted in improved mitochondrial stability at 18hrs when compared to controls (Fig 3E). Mitochondria from LR treated animals could only absorb 357 ± 24 nmol Ca^2+^/mg before developing MPT, whereas mitochondria from AVP treated animals behaved like Shams and could tolerate a significantly higher Ca^2+^ load (406 ± 26 nmol/mg, p < 0.05).

### Oxidant damage and histology

**Fig 4:**
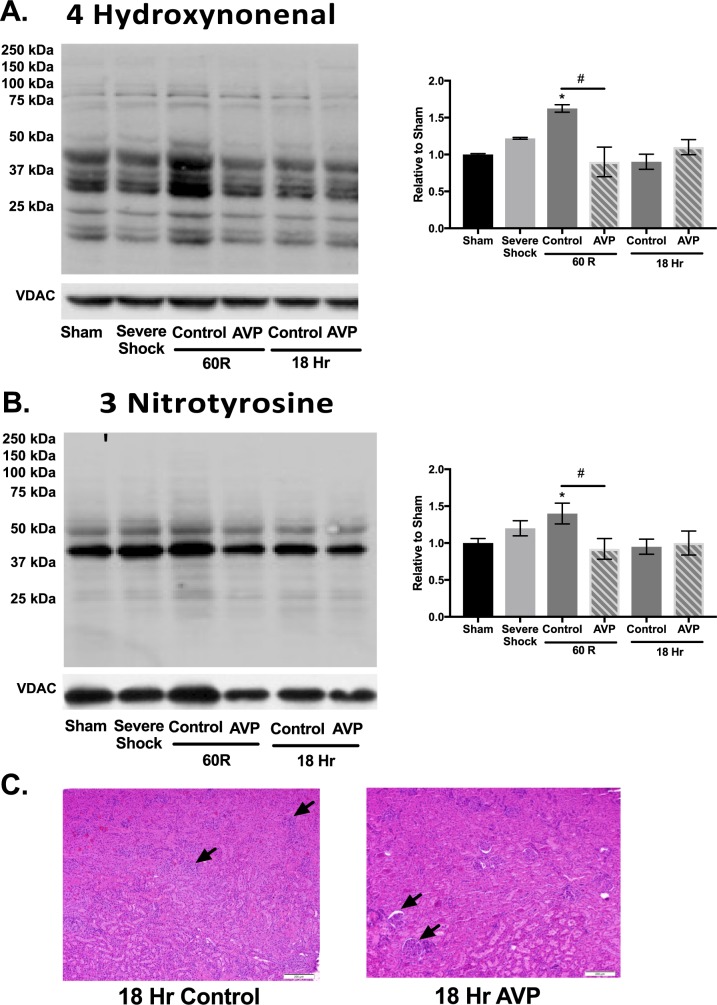
AVP supplementation during hemorrhagic shock is associated with decreased reactive species damage and preserved histologic architecture. Kidney protein lystates (20μg) were analyzed by SDS-Page using antibodies directed toward 4-hydroxynonenal (HNE) and 3-nitrotyrosine as a measure of oxidative damage. Kidney samples were sectioned, stained with hematoxylin and eosin, and imaged at 10X. (**A, B**) AVP significantly decreased oxidative damage following resuscitation (60R). (**C**) AVP also preserved renal architecture with normal glomeruli (depicted by black arrows) observed 18 hours post-resuscitation. N = 6 animals per time point. Values are mean ± SEM. *N* = 6–7 per time point. Data were analyzed using one-way ANOVA with a post hoc Tukey’s test. Histologic grading was analyzed using a Mann Whiney U test. *p<0.05 *vs*. Sham; #*p*<0.05 Control *vs*. AVP treated.

The use of AVP significantly decreased acute kidney injury as measured by oxidant damage (Fig 4A and 4B). Both the degree of lipid peroxidation and protein nitration, as measured by antibodies to 4-hydoxynonenal and 3-nitrotyrosine respectively, were significantly higher 60R in animals treated with only LR. No difference in oxidant damage was noted at 18hrs between groups and values had returned to baseline. Finally, resuscitation with AVP was associated with significantly improved histologic architecture at 18hrs using the median EGTI score (Control; 5 (range 2–6) vs AVP; 2 (range 1–3). p<0.05) (Fig 4C).

## Discussion

Hemorrhagic shock is a common, and potentially preventable, cause of death following trauma. Although hemorrhage control and blood volume replacement are the mainstays of therapy, targeting the pathophysiology of decompensated shock could lead to improved outcomes. AVP, a vasoactive hormone that is stored and subsequently secreted from the posterior pituitary during acute hypotension, plays a critical role in maintaining hemodynamic stability during hemorrhagic shock.[12] Here, we demonstrate that both pituitary and serum levels of AVP decrease during decompensated hemorrhagic shock and remain depressed despite persistent hypotension. Moreover, this decline in AVP correlated with the development of catecholamine-resistant shock. Supplementing exogenous AVP during resuscitation significantly improved blood pressure and renal function immediately post-resuscitation. As such, AVP may provide a useful adjunct following severe hemorrhagic shock.

The vasopressor effect of posterior pituitary extracts, now known as arginine vasopressin (AVP), are well known and were first described in 1895.[34] Since then, it has been appreciated that serum AVP levels can increase modestly in response to increased osmolality, but can rapidly increase 20 to 200 fold in states of shock. Once vasopressin stores have been released, however, it may take up to 2 hours to synthesize, transfer and subsequently secrete new AVP protein.[35, 36]

The actions of AVP are mediated by 5 different G-protein coupled receptors (V1, V2, V3, oxytocin, and purinergic) with different tissue specificity and intracellular pathways.[37] During hemorrhagic shock, V1 receptors, in particular, play a pivotal role in maintaining cardiovascular homeostasis. V1 receptors are found primarily on vascular smooth muscle, but are also expressed in high density in the renal medulla. AVP causes vasoconstriction by increasing intracellular calcium via the phosphatidyl- inositol-biphosphonate pathway. In the renal medulla, however, AVP selectively constricts the efferent but not the afferent arterioles, thus leading to an increase in glomerular filtration.[38]

Although serum AVP levels are initially elevated during shock, levels can fall inappropriately during prolonged shock states and may contribute to the development of decompensated shock.[11, 13] In our model, we observed that serum AVP levels fell dramatically during extended hypotension. Importantly, restoring AVP levels during resuscitation returned the MAP to normal values whereas high-dose catecholamines had no effect on blood pressure. These data support the concept of relative AVP deficiency in hemorrhagic shock as previously described by Morales et al. [13] Using a canine model of acute blood loss, these investigators found that AVP levels fell 10 fold during prolonged hypotension. These inappropriately low AVP values correlated with the development of refractory hypotension that could only be rescued with supplemental AVP. Similarly, we also observed the development of catecholamine resistance in our model. Although we did not measure AVP at the initiation of shock, we did observe a 50% reduction in serum AVP levels from the point of Decompensation to Severe Shock despite persistent hypotension.

Although increased consumption of AVP may contribute to low serum AVP levels in hemorrhagic shock; our data supports hypothesis that impaired production may also play a role.[39] We observed a progressive depletion of pituitary AVP stores with prolonged hemorrhagic shock that persisted for at least 18 hours post-resuscitation. Importantly, while there was a trend toward improved pituitary stores with AVP supplementation during resuscitation, this trend was not maintained 18 hours later.

Given we could not restore the MAP with other adrenergic agents, we cannot definitively determine if AVP has direct cellular benefit independent of its impact on blood pressure shock. Nonetheless, a number of *in vitro* studies support our findings that AVP positively impacts mitochondrial function both acutely and at 18 hours post resuscitation. In isolated hepatocytes, AVP increases cytosolic as well as mitochondrial calcium, which in turn activates Ca^2+^ dependent Kreb cycle dehydrogenases and increases the level of NADH.[20, 40] The elevation in NADH stimulates CI dependent respiration.[20] Additionally, because AVP increases the mitochondrial Ca^2+^ concentration, there is a shift in the membrane potential that favors the proton motive force and subsequent ATP production.[21] AVP also decreases apoptosis following anoxia. In isolated hepatocytes, Hoek and colleagues noted that although vasopressin increases intracellular calcium levels, it prevented excessive mitochondrial swelling and the transition to mitochondrial permeability transition–a Ca^2+^ mediated event that triggers apoptosis.[22] Similarly, our results demonstrate that AVP supplementation during resuscitation significantly preserved CI-dependent respiration and electron transfer. The use of AVP also decreased the generation of mitochondrial ROS, a byproduct of impaired CI activity.[41] Resuscitation with AVP was also decreased renal oxidant damage as measured by lipid peroxidation and protein nitrosylation. Lastly, mitochondria isolated from AVP-treated rats demonstrated increased stability and were less susceptible to mitochondrial permeability transition. Taken together, the use of AVP during resuscitation appears to prevent the development of shock-induced mitochondrial dysfunction.

AVP also significantly ameliorated shock-induced acute kidney injury (AKI) immediately following resuscitation and resulted in decreased serum neutrophil gelatinase-associated lipocalin (NGAL). NGAL is a 25 kDa protein covalently bound to matrix metalloproteinase-9 in neutrophils that is markedly induced following epithelial injury.

Elevations in NGAL precede changes in serum creatinine and can be used to diagnose AKI up to 48 hours prior to a clinical change in creatinine or urine output.[42, 43] Interestingly, although AVP preserved renal architecture at 18 hours, there were no differences between groups in terms of NGAL or oxidant damage, suggesting that the benefit of supplementing AVP may be transient. Given the half-life of AVP is only 10–35 minutes [35], it would not be surprising if the impact of exogenous AVP waned; with low serum levels contributing to the persistent hypotension we observed at 18 hours.

Although encouraging, our study has several limitations. First, we only investigated the use of high dose AVP given at a single time point. While high dose vasopressin has been shown to improve survival in a lethal model of hemorrhagic shock in swine [44, 45], its long-term benefit remains unknown and alternative dosing regimens, including the use of a continuous infusion targeting a physiologic endpoint such as MAP, may provide a better supplementation strategy. Second, we only evaluated the impact of AVP on one tissue type. Given the variety of different vasopressin receptors as well as the susceptibility of different tissues to ischemia, it is possible that AVP may have tissue-specific effects. Finally, despite our attempt to control for AVP’s vasoconstrictive properties, we were not able to restore a “normal” MAP using either norepinephrine or epinephrine and, therefore, cannot definitively determine the direct impact of AVP on cellular function.

## Conclusion

Decompensated hemorrhagic shock results in a progressive depletion of pituitary AVP stores, as well a decline in serum AVP levels. Supplementing exogenous AVP during resuscitation improves blood pressure, preserves renal mitochondrial function, and mitigates early acute kidney injury. While further work is needed to determine the optimal dose and timing, AVP appears to be a beneficial adjunct during the resuscitation of severe shock.

## Supporting information

S1 Data Data supporting the findings reported in this manuscript can be found in the supplemental file. (XLSX)
